# Hypoxia-inducible gene 2 promotes the immune escape of hepatocellular carcinoma from nature killer cells through the interleukin-10-STAT3 signaling pathway

**DOI:** 10.1186/s13046-019-1233-9

**Published:** 2019-05-29

**Authors:** Chuanbao Cui, Kaiwen Fu, Lu Yang, Shuzhi Wu, Zuojie Cen, Xingxing Meng, Qiongguang Huang, Zhichun Xie

**Affiliations:** 1 Department of Epidemiology, Guangxi Medical University, No. 22 Shuangyong Road, Nanning, 530021 Guangxi Zhuang Autonomous Region People’s Republic of China; 2 Department of Pathology, Chongqing University Cancer Hospital, Chongqing, People’s Republic of China

**Keywords:** Hypoxia-inducible gene 2, Hepatocellular carcinoma, Nature killer cells, IL-10, STAT3

## Abstract

**Background:**

The study examines the expression and function of hypoxia-inducible gene 2 (HIG2) in hepatocellular carcinoma (HCC) tissues and cells.

**Methods:**

Forty patients with HCC were included in the study. Bioinformatic analysis was used to analyze the clinical relevance of *HIG2* expression in HCC tissue samples. Immunohistochemistry was employed to determine the expression of target proteins in tumor tissues. Hepatic HepG2 and SMMC-7721 cells were transfected with *HIG2*-targeting siRNA with Lipofectamine 2000. qRT-PCR was carried out to determine gene expression levels, while Western blotting was used to determine protein expression. A CCK-8 assay was performed to detect proliferation of cells, while migration and invasion of cells were studied by Transwell assay. Flow cytometry was carried out to detect surface markers and effector molecules in Nature killercells, as well as the killing effect of NK cells.

**Results:**

HIG2 expression was upregulated in HCC. Silencing of *HIG2* suppressed HCC cell migration and invasion. The killing effect of NK cells on HCC cells was enhanced after *HIG2* was silenced in HCC cells. Conditioned media from *HIG2*-silenced SMMC-7721 cells inhibited the phenotype and function of NK cells. HCC cells with silenced expression of *HIG2* modulated the activity of NK cells via STAT3. *HIG2* promoted the evasion of HCC cells from killing by NK cells through upregulation of IL-10 expression.

**Conclusion:**

The study demonstrates that HIG2 activates the STAT3 signaling pathway in NK cells by promoting IL-10 release by HCC cells, thereby inhibiting the killing activity of NK cells, and subsequently promoting the recurrence and metastasis of HCC.

## Background

Hepatocellular carcinoma (HCC) is one of the most common malignant tumors in the world, and its incidence is higher in men than in women [1, 2]. In China, the incidence of HCC ranks fourth among all malignant tumors, and its mortality rate ranks second [3]. At present, surgical resection is still the first choice for treating HCC, but the prognosis is poor after radical surgery, with a 5-year survival rate of approximately 16% [4]. Recurrence and metastasis of HCC are key factors that limit clinical outcomes. It has been reported that the recurrence and metastasis of HCC are complex processes, which mainly include inactivation or mutation of tumor-suppressor genes and abnormal activation of oncogenes [5, 6]. The molecular mechanism of recurrence and metastasis of HCC remains unclear. Therefore, studying the mechanism of HCC at the molecular level and finding effective therapeutic measures have become of great scientific and clinical importance in HCC.

Hypoxia-inducible gene 2 (*HIG2*), which is located at q32.1 of human chromosome 7, is a newly discovered gene that can be induced by hypoxia and lack of glucose. With a complete length of 3.4 kb, it contains two exons and one intron [7, 8]. Expression of *HIG2* is induced in hypoxic environments, and *HIG2* has been proven to be a target gene of hypoxia-inducible factor-1 (HIF-1) [9]. It has been reported that HIG2 is a new type of lipid droplet (LD) protein, which stimulates the accumulation of lipids in cells [10]. In recent years, the role of the *HIG2* gene in the occurrence and development of tumors has garnered significant research interest. Studies have shown that *HIG2* plays an important role in the development and progression of renal cell carcinoma, cell lymphoma, epithelial ovarian cancer, transparent cell adenocarcinoma, and uterine cancer [11, 12].

Innate immunity is the first line of defense against microbial infection and cancers [13]. Natural killer cells are the most important natural immune cells, and have powerful tumor-killing functions. Natural killer (NK) cells are derived from the bone marrow, and account for 10–18% of peripheral blood mononuclear lymphocytes [14]. NK cells can be phenotyped as CD3^−^CD56^+^ lymphocytes. Animal and clinical experiments have confirmed that the number and activity of NK cells are directly related to tumorigenesis and prognosis [15]. Higher number and activity of NK cells usually correspond to stronger suppression of tumors. Tumor tissues are infiltrated by a large number of NK cells, and tumor cells with high metastatic potential need to escape immune surveillance before metastasis can occur [5]. However, the activity and function of NK cells that infiltrate tumor tissues are inhibited in varying degrees. If the inhibition of NK cells by the tumor microenvironment can be relieved, the killing effect of NK cells on tumors can be restored [16]. As the main component of tumors, tumor cells can have a strong regulatory effect on the tumor microenvironment [17]. However, this underlying mechanism still needs to be further explored. In the present study, we examine the expression and function of *HIG2* in HCC tissues and cells and investigate the effect of *HIG2* on HCC cell regulation of the immunological function of NK cells.

## Materials and methods

### Patients

A total of 40 patients with HCC who underwent surgical resection at Chongqing Cancer Hospital between January 2016 and December 2017 were included in the study (29 males and 11 females; age range, 32–55 years; mean age, 43.6 years). None of the patients had a history of any other types of malignant tumors or chemoradiotherapy. Among the patients, 22 cases had lymph node metastasis and 18 cases had no lymph node metastasis. According to the 2003 TNM staging standards by the Union for International Cancer Control, 11 cases were Stage I, 16 cases were Stage II, 5 cases were Stage III, and 8 cases were Stage IV. HCC tissues and tumor-adjacent tissues were resected from all patients and included in the experimental and control groups, respectively. All procedures performed in the current study were approved by the Ethics Committee of Chongqing Cancer Hospital. A written informed consent was obtained from all patients or their families.

### Bioinformatics

Bioinformatic analysis was used to analyze the clinical relevance of *HIG2* gene expression in HCC tissues. We utilized the Gene Expression Profiling Interactive Analysis (GEPIA) database (http://gepia.cancer-pku.cn/) to assess the correlation between *HIG2* expression and 5-year overall survival, and disease-free survival of HCC patients.

### Immunohistochemistry

Freshly resected liver tissues were fixed overnight with 4% paraformaldehyde and paraffin-embedded before being sectioned at 4 μm. Paraffin sections were dewaxed at 67 °C for 2 h before being washed three times with phosphate-buffered saline (PBS) for 3 min each time. Dewaxed tissue slices were boiled for 20 min in citrate buffer (pH = 6.0) and cooled to room temperature. After washing with PBS twice, slides were each covered with 3% H_2_O_2_ and then incubated at 37 °C for 10 min. After washing with PBS, slides were each covered with 100 μl of HIG2 and IL-10 primary antibodies (1:50 dilution for both) and incubated at room temperature for 2 h. After washing with PBS, slides were each covered with 100 μl of polymer enhancer before incubation at room temperature for 20 min. After washing with PBS, slides were each covered with 100 μl of enzyme-labeled anti-mouse / rabbit polymers before incubation at room temperature for 1 h. After washing with PBS, slides were each covered with 1 drop of diaminobenzidine (DAB) and observed under a microscope after 5 min. Slides were then stained with hematoxylin, differentiated with 0.1% HCl, and washed with water. The slides were then dehydrated using an increasing alcohol gradient, vitrificated by dimethylbenzene, and fixed by neutral balata. After drying, the slice was observed under a light microscope.

### Cells

Hepatic HepG2 and SMMC-7721 cells (Cell Bank, Chinese Academy of Sciences, Shanghai, China) were cultured in DMEM supplemented with 10% fetal bovine serum (FBS), 100 IU/ml penicillin, and 100 IU/ml streptomycin (all reagents from Thermo Fisher Scientific, Waltham, MA, USA) at 37 °C, 5% CO_2_ and 70% humidity. The cells were passaged every 3 days, and those in logarithmic growth were collected for further assays.

One day before transfection, HepG2 and SMMC-7721 cells (2 × 10^5^) in logarithmic growth were seeded in 24-well plates containing antibiotics-free DMEM supplemented with 10% FBS. Cells were transfected at 70% confluence. In the first vial, 1.5 μL siR-NC or siR-HIG2 (20 pmol/μL; Hanbio Biotechnology Co., Ltd., Shanghai, China) was mixed with 50 μl Opti Mem medium (Thermo Fisher Scientific). In the second vial, 1 μL Lipofectamine 2000 (Thermo Fisher Scientific) was mixed with 50 μl Opti Mem medium. After a 5-min incubation, the two vials were combined and the mixture was incubated at room temperature for 20 min. The mixtures were then added onto cells in the respective groups. Six hours later, the medium was replaced with DMEM containing 10% FBS. After cultivation for 48 h, the cells were collected for further assays.

To isolate peripheral blood mononuclear lymphocytes, 3 ml peripheral blood was gently added onto the surface of 3 ml Ficoll solution (MagniSort™ Mouse NK cell Enrichment Kit; Thermo Fisher Scientific, Waltham, MA, USA), followed by centrifugation at 650×g and 4 °C for 20 min. The middle layer containing lymphocytes was carefully transferred to a new 15 ml tube. The separated lymphocytes were mixed with PBS up to a maximum volume of 10 ml and centrifuged at 250 x g and 4 °C for 10 min. The cell pellet was resuspended in 5 ml PBS and then centrifuged at 250 x g for 10 min. Isolated peripheral blood mononuclear lymphocytes were resuspended in 1 ml of 1X BD IMag buffer. Ten microliters of the suspension were then mixed with 190 μl PBS. Then, 5 μl biotinylated human NK cell concentrate was added and incubated in the dark at room temperature for 15 min. Then, 1.8 ml 1X BD buffer was added to remove biotin, followed by centrifugation at 300 x g for 7 min. Finally, the cells were resuspended in 500 μl of 1X BD buffer, followed by addition of equal volume of beads solution. After incubation in the dark for 30 min, the mixture was mixed gently and placed on a magnet for 7 min. The supernatant was then transferred to a new Eppendorf tube, and NK cells were obtained. NK cells were cultured in RPMI-1640 medium supplemented with 10% FBS and 100 IU IL-2 at 37 °C and 5% CO_2_ for 48 h before use.

### Quantitative real-time polymerase chain reaction (qRT-PCR)

Tissue samples (100 mg) were flash frozen in liquid nitrogen, ground, and then lysed with 1 ml TRIzol following the manufacturer’s manual (Thermo Fisher Scientific, Waltham, MA, USA). Cells (1 × 10^6^) were directly lysed with 1 ml TRIzol. Total RNA was extracted using phenol chloroform. The concentration and quality of RNA was measured using ultraviolet spectrophotometry (Nanodrop ND2000, Thermo Scientific, Waltham, MA, USA). cDNA was then obtained by reverse transcription of 1 μg RNA and stored at − 20 °C. Reverse transcription of mRNA was performed using TIANScript II cDNA First Strand Synthesis Kit (Tiangen, Beijing, China), and reverse transcription of miRNA was carried out using miRcute miRNA cDNA First Strand Synthesis Kit (Tiangen, Beijing, China). SuperReal PreMix (SYBR Green) qRT-PCR kit (Tiangen, Beijing, USA) was used to detect mRNA expression of *HIG2,* using *GAPDH* as an internal reference. The primer sequences of *HIG2* were 5′- ACGAGGGCGCTTTTGTCTC − 3′ (forward) and 5′- AGCACAGCATACACCAGACC − 3′ (reverse). The primer sequences of *GAPDH* were 5′- CGGAGTCAACGGATTTGGTCGTAT − 3′ (forward) and 5′- AGCCTTCTCCATGGTGGTGAAGAC − 3′ (reverse). The reaction (20 μl) was composed of 10 μl SYBR Premix EXTaq, 0.5 μl upstream primer, 0.5 μl downstream primer, 2 μl cDNA and 7 μl ddH_2_O. PCR conditions were: initial denaturation at 95 °C for 10 min; denaturation at 95 °C for 1 min and annealing at 60 °C for 30 s (40 cycles; iQ5; Bio-Rad, Hercules, CA, USA). The 2^-ΔΔCq^ method [18] was used to calculate the expression of *HIG2* mRNA relative to GAPDH. Each sample was tested in triplicate.

### CCK-8 assay

HepG2 and SMMC-7721 cells were seeded at a density of 2000/well in 96-well plates. At 0, 24, 48, and 72 h, 20 μl CCK-8 reagent (5 g/L; Beyotime, Shanghai, China) was added to the cells. At the designated time points, 150 μl CCK-8 reaction solution was added, and the cells were incubated at 37 °C for 2 h. Then, the absorbance of the cells in each well was measured at 490 nm for plotting cell proliferation curves. Each group was tested in three replicate wells, and the values were averaged.

### Transwell assay

Growth factor-depleted Matrigel invasion chambers (BD Biosciences, Franklin Lakes, NJ, USA) were used to measure cell invasion. Matrigel was thawed at 4 °C overnight and diluted with serum-free DMEM (dilution 1:2). The mixture (50 μl) was evenly distributed into the upper chamber (Merck Millipore, Billerica, MA, USA) and incubated at 37 °C for 1 h. After solidification, 1 × 10^5^ cells from each group were seeded into the upper chamber containing 200 μl of serum-free DMEM. In addition, 500 μl DMEM supplemented with 10% fetal bovine serum was added to the lower chamber. After 24 h, the chamber was removed, and the cells in the upper chamber were wiped off. After being fixed with 4% formaldehyde for 10 min, the membrane was stained using Giemsa method for microscopic observation of five random fields (200×). For migration array, the tumor cells were seeded onto the upper chamber (Merck Millipore, Billerica, MA, USA) without matrigel, and the rest of the steps were the same as the invasive array. The number of motile cells was calculated to evaluate cell invasion and migration. All procedures were carried out on ice with pipetting tips being cooled to 4 °C.

### Western blotting

Before lysis, tissues (100 mg) were ground into powder, and cells (1 × 10^6^) were trypsinized and collected. Tissue samples or cells were then lysed with chilled radio-immunoprecipitation assay (RIPA) lysis buffer (600 μl; 50 mM Tris-base, 1 mM EDTA, 150 mM NaCl, 0.1% sodium dodecyl sulfate, 1% TritonX-100, 1% sodium deoxycholate; Beyotime Institute of Biotechnology, Shanghai, China) for 30 min on ice. The mixture was centrifuged at 12,000 rpm and at 4 °C for 10 min. The supernatant was used to determine protein concentration by bicinchoninic acid (BCA) protein concentration determination kit (RTP7102, Real-Times Biotechnology Co., Ltd., Beijing, China). The samples were then mixed with 5× sodium dodecyl sulfate loading buffer before denaturation in boiling water bath for 10 min. Afterwards, the samples (20 μg) were subjected to 10% sodium dodecyl sulfate-polyacrylamide gel electrophoresis at 100 V. The resolved proteins were transferred to polyvinylidene difluoride membranes on ice (250 mA, 1 h) and blocked with 5% skimmed milk at room temperature for 1 h. The membranes were then incubated with mouse anti-human HIG2 (1:1000; ab78349; Abcam, Cambridge, UK), rabbit anti-human CREB (1:800; ab32515; Abcam), rabbit anti-human NF-kB p65 (1:1000; ab16502; Abcam), mouse anti-human STAT3 (1:1000; ab119352; Abcam), rabbit anti-human STAT1 (1:1000; ab30645; Abcam), rabbit anti-human STAT4 (1:800; ab235946; Abcam), rabbit anti-human STAT5 (1:800; ab16276; Abcam), rabbit anti-human STAT6 (1:800; ab44718; Abcam), mouse anti-human p53 (1:800; ab90363; Abcam) or GAPDH (1:4000; ab70699; Abcam) monoclonal primary antibodies at 4 °C overnight. After extensive washing with phosphate-buffered saline with Tween 20 5 times for 5 min each time, the membranes were incubated with goat anti-mouse horseradish peroxidase-conjugated secondary antibody (1:4000; ab6789; Abcam, Cambridge, UK) for 1 h at room temperature before washing with phosphate-buffered saline with Tween 20 5 times for 5 min each time. Membranes were then developed with enhanced chemiluminescence detection kit (Sigma-Aldrich, St. Louis, MO, USA) for imaging. Image lab v3.0 software (Bio-Rad, Hercules, CA, USA) was used to acquire and analyze imaging signals. Each target protein was quantified relative to GAPDH protein levels.

### Flow cytometry

According to the manufacturer’s manual, 1 × 10^5^ NK cells were suspended in 100 μl DMEM medium before addition of fluorescence-labelled, activated receptors (p30, CD16, p46, and NKG2D), and inhibitory receptors (158b and NKG2A). The cells were then incubated at room temperature in the dark for 15 min before examination by flow cytometry. For the detection of effector molecules, NK cells were pre-stained with CD3 and CD56 antibodies, followed by addition of fluorescence-labelled GZMB, Perforin, TNF-α, and INF-γ antibodies. After incubation in the dark at room temperature for 15 min, the cells were washed 3 times with cold PBS before centrifugation at 600×g for 5 min to collect the cells. After resuspending the cells in 200 μl cold PBS, flow cytometry was performed.

### Determination of tumor-killing effect of NK cells by flow cytometry

NK cells (2 × 10^4^) were mixed with SMMC-7721 or HepG2 cells at a ratio of 1:4, and cultured at 37 °C and 5% CO_2_ overnight. The cell density was then adjusted to 1 × 10^5^/100 μl and subjected to flow cytometry analysis using the ANXN V FITC APOPTOSIS DTEC KIT I (BD Biosciences, Franklin Lakes, NJ, USA) following the manufacturer’s manual for the detection of apoptosis. Cells with ANNEXIN V-positive values were early apoptotic cells, those with PI-positive values were necrotic cells, and those with double positive values were late apoptotic cells.

### Lactate dehydrogenase (LDH) assay

Cells were seeded in 24-well plates at a density of 2 × 10^5^ cells/well, and incubated with serum from healthy subjects, sepsis patients or septic shock patients for 24 h. Then, the medium was replaced with fresh medium, followed by incubation for 12 h. The supernatant was collected and centrifuged at 12,000×g for 10 min. Afterwards, 120 μl of supernatant was used for LDH assay following the manufacturer’s manual (Beyotime, Shanghai, China).

### Tumorigenesis assay in nude mice

For performing the tumorigenesis assay in vivo, female BALB/c-nu mice (5–6 weeks of age, 16–20 g) were purchased and kept in barrier facilities on a 12 h light/dark cycle. All experimental procedures were approved by the Institutional Animal Care and Use Committee of Guangxi Medical University. Briefly, BALB/c-nu mice were injected with 1 × 10^6^ of the indicated cells under the armpit (tumor cells were suspended in 200 μl sterile PBS). Six weeks later, all mice were euthanized, and tumors were dissected and sectioned (4 μm in thickness), followed by H&E staining or IHC.

### Metastasis assay in nude mice

For pulmonary metastasis assays, the nude mice were divided into 2 groups, siR-NC and siR-HIG2. Two million SMMC-7721 cells transfected with siR-HIG2 or siR-NC were suspended in 200 μl phosphate-buffered saline for each mouse. The indicated tumor cells were injected into nude mice (6 per group, 5-week-old) through the lateral tail vein. After 6 weeks, the mice were euthanized and each lung was dissected and fixed with phosphate-buffered neutral formalin before paraffin embedment. The paraffin blocks were then cut into five sections and stained with H&E. We then observed the sections under a light microscope for calculating the metastatic nodules.

### Dual-luciferase reporter assay

To investigate whether CREB protein could directly bind to the promoter region of IL-10, dual-luciferase reporter assay was performed in vitro. In brief, the promoter sequence of the *IL-10* gene was predicted in silico (http://biogrid-lasagna.engr.uconn.edu) and amplified by qRT-PCR. The primers were as follows: 5′-AGGAGAAGTCTTGGGTATTCATCC-3′ (forward) and 5′-AAGCCCCTGATGTGTAGACC-3′ (reverse). The plasmid harboring the TSS sequence of CREB (pcDNA3.1-CREB) was constructed by Genechem Co. Ltd. (Shanghai, China). The promoter sequence of *IL-10* was cloned into the luciferase reporter plasmid pGL6 (Beyotime, Beijing, China) that contained XhoI or HindIII restriction sites. 293 T cells were transfected with the reporter plasmid together with pcDNA3.1-CREB using liposome method. After 24 h of incubation, the cells from each group were lysed according to the manufacturer’s instructions (Beyotime, Beijing, China). Luminescence intensity was recorded by a GloMax 20/20 luminometer (Promega Corp., Fitchburg, WI, USA). Luminescence activity of Renilla luciferase was used as the internal reference, and cell luminescence values in each group were statistically analyzed.

### Statistical analysis

Continuous variables are represented by mean ± standard deviation. The comparison between two groups was performed by using student’s *t* test. A *p*-value less than 0.05 was considered statistically significant. ANOVA followed by a post hoc multiple comparisons test was used for the comparison of multiple groups. All experiments were repeated three times.

## Results

### *HIG2* expression is upregulated in HCC

**Fig. 1:**
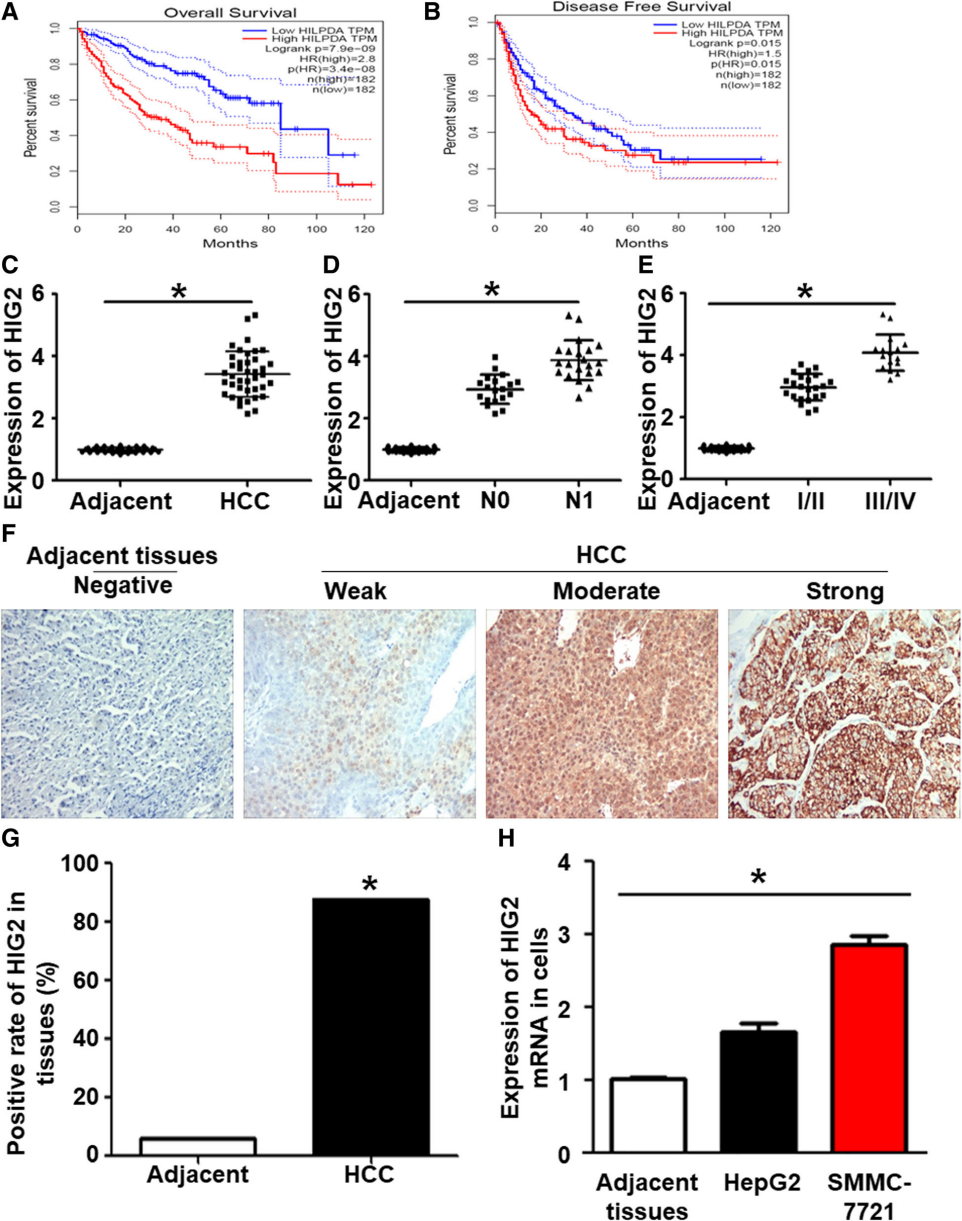
Expression of HIG2 in HCC tissues and cells. **a** Overall survival of HCC patients with different levels of *HIG2* expression. **b** Disease-free survival of HCC patients with different levels of *HIG2* expression. **c** *HIG2* mRNA expression in HCC tissues in comparison with tumor-adjacent tissues. *P < 0.05. **d** *HIG2* mRNA expression in HCC tissues from patients with or without lymph node metastasis in comparison to tumor-adjacent tissues. *P < 0.05. **e** *HIG2* mRNA expression in HCC tissues from patients with TNM Stage I/II or III/IV disease in comparison to tumor-adjacent tissues. *P < 0.05. **f** Immunohistochemical analysis of HIG2 expression in HCC tissues and tumor-adjacent tissues. **g** High incidence of HIG2 expression in HCC tissues in comparison to tumor-adjacent tissues. *P < 0.05. **h** Expression of *HIG2* mRNA in HepG2 and SMMC-7721 cells in comparison to tumor-adjacent tissues. *P < 0.05

The GEPIA database was used to perform a preliminary assessment of the association between the expression of *HIG2* gene in HCC tissues and prognosis of HCC. The search results showed that the level of the *HIG2* gene in HCC tissues was significantly higher than that in tumor-adjacent tissues (Fig. 1a). Postoperative survival analysis showed that the 5-year survival and disease-free survival rates of HCC patients with high expression of *HIG2* were lower than those of HCC patients with low expression of *HIG2* (Fig. 1b and c). Our data showed that *HIG2* mRNA expression in HCC tissues was significantly higher than that in tumor-adjacent tissues (*P* < 0.05) (Fig. 1d). In addition, *HIG2* expression in tumor tissues from HCC patients with lymph node metastasis was significantly higher than that from HCC patients without lymph node metastasis (*P* < 0.05) (Fig. 1e). *HIG2* expression in tumor tissues from HCC patients with TNM Stage III/IV disease was significantly higher than that in tumor tissues from HCC patients with TNM Stage I/II disease (*P* < 0.05) (Fig. 1f). Immunohistochemistry showed that HIG2 expression was detected in the majority of the tested HCC tissues (35/40), and only in a small number of tumor-adjacent tissues (2/40) (Fig. 1g and h). Consistently, the expression of *HIG2* mRNA in HepG2 and SMMC-7721 cells was significantly higher than that in tumor-adjacent tissues (*P* < 0.05; Fig. 1i). These results suggest that *HIG2* expression is upregulated in HCC.

### Silencing of *HIG2* suppresses HCC cell migration and invasion in vitro and in vivo

**Fig. 2:**
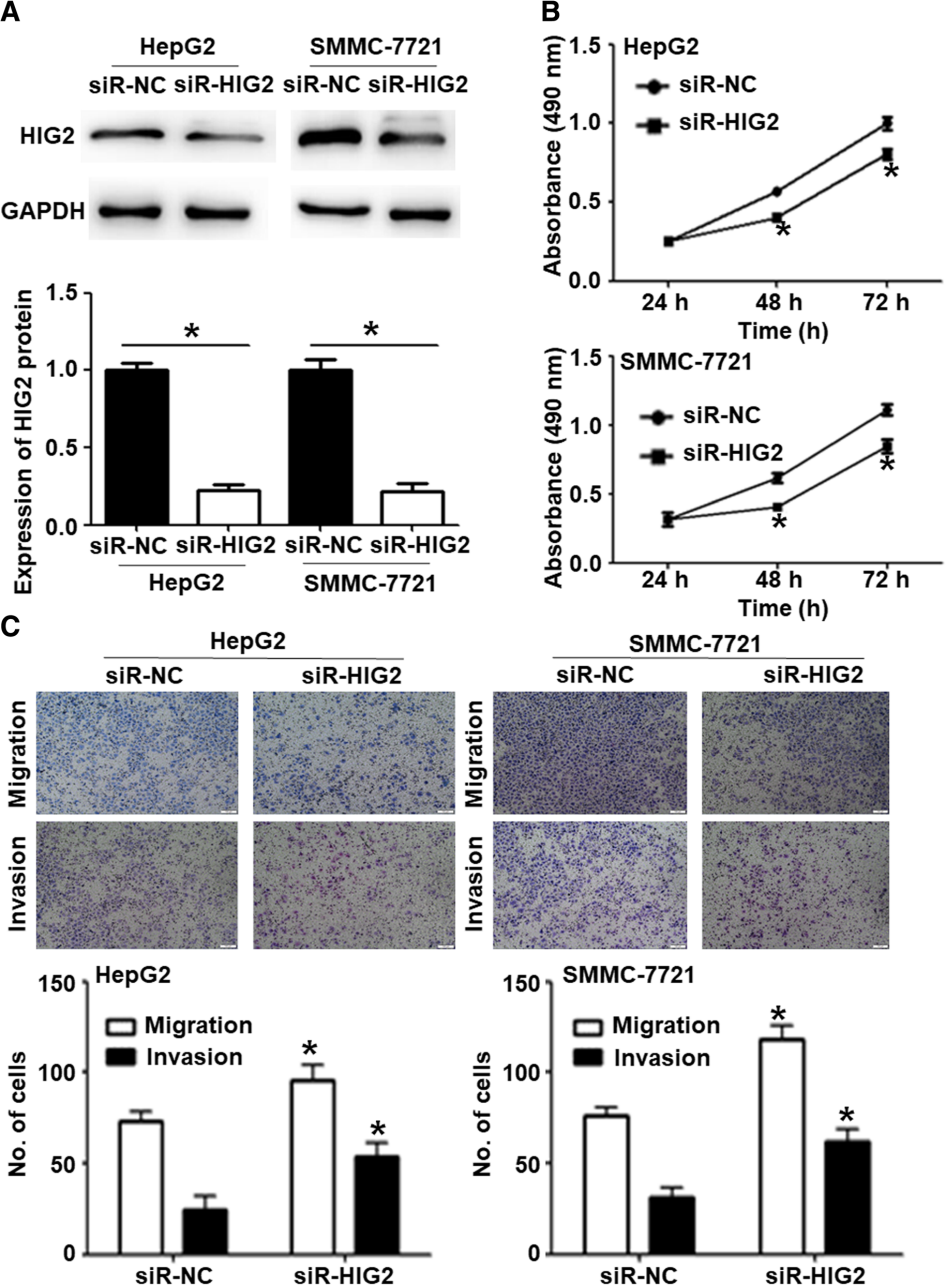
Effect of silencing *HIG2* on HCC cell migration and invasion in vitro. **a** HIG2 protein expression in HepG2 and SMMC-7721 cells transfected with siR-HIG2 or siR-NC, as determined by Western blotting. *P < 0.05. **b** Proliferation of HepG2 and SMMC-7721 cells transfected with siR-HIG2 or siR-NC, as determined by CCK-8 assay. *P < 0.05 compared with siR-NC at the same time points. **c** Migration and invasion of HepG2 and SMMC-7721 cells transfected with siR-HIG2 or siR-NC, as determined by Transwell assay. *P < 0.05 compared with the respective siR-NC group

**Fig. 3:**
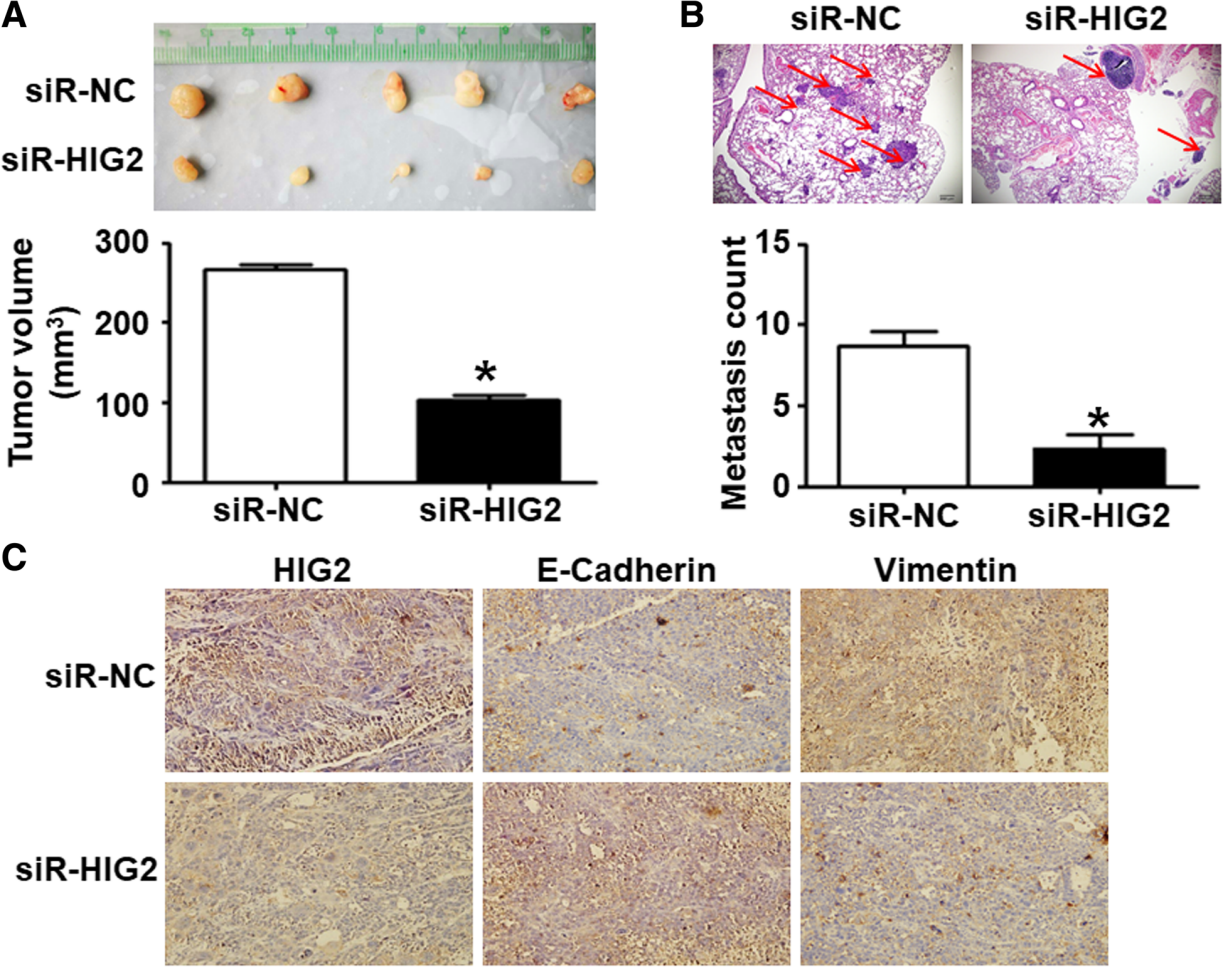
HCC tumors in nude mice induced by *HIG2-*silenced SMMC-7721 cells. **a** Volume of tumors in nude mice induced by SMMC-7721 cells transfected with siR-HIG2 or siR-NC. *P < 0.05 compared with the siR-NC group. **b** Lung metastasis count in siR-HIG2 and siR-NC groups. *P < 0.05 compared with siR-NC group. **c** Expression of the epithelial marker E-Cadherin and the interstitial marker Vimentin in tumors from the siR-HIG2 and siR-NC groups, as determined by immunohistochemistry

To further study the function of the *HIG2* gene in HCC, we used siR-HIG2 to downregulate the expression of *HIG2* in HepG2 and SMMC-7721 cells. Western blotting showed that HIG2 protein levels in HepG2 and SMMC-7721 cells transfected with siR-HIG2 were significantly lower than that in the cells transfected with siR-NC (*P* < 0.05) (Fig. 2a). CCK-8 assay showed that the proliferation of HepG2 and SMMC-7721 cells transfected with siR-HIG2 was significantly reduced in comparison to the proliferation of cells transfected with siR-NC (*P* < 0.05) (Fig. 2b). In addition, Transwell assay showed that the number of migratory HepG2 and SMMC-7721 cells in the siR-HIG2 group was significantly lower than those in the siR-NC group (*P* < 0.05) (Fig. 2c). We then generated nude mouse models of tumor formation and lung metastasis using SMMC-7721 cells. The results showed that the mean volume of tumors induced by SMMC-7721 cells transfected with siR-HIG2 were significantly smaller than that of tumors induced by SMMC-7721 cells transfected with siR-NC (*P* < 0.05) (Fig. 3a). Additionally, a smaller number of metastatic foci were observed in the siR-HIG2 group in comparison to the siR-NC group (*P* < 0.05) (Fig. 3b). Immunohistochemical analysis showed that the epithelial marker E-Cadherin was upregulated in tumors from the siR-HIG2 group, while the expression of the interstitial marker Vimentin was downregulated in tumors from the siR-HIG2 group. This suggests that the epithelial-to-mesenchymal transition (EMT) in the siR-HIG2 group was enhanced (Fig. 3c). The results indicate that silencing of *HIG2* suppresses HCC cell migration and invasion in vitro and in vivo.

### The killing effect of NK cells on HepG2 and SMMC-7721 cells is enhanced after *HIG2* silencing in HepG2 and SMMC-7721 cells

**Fig. 4:**
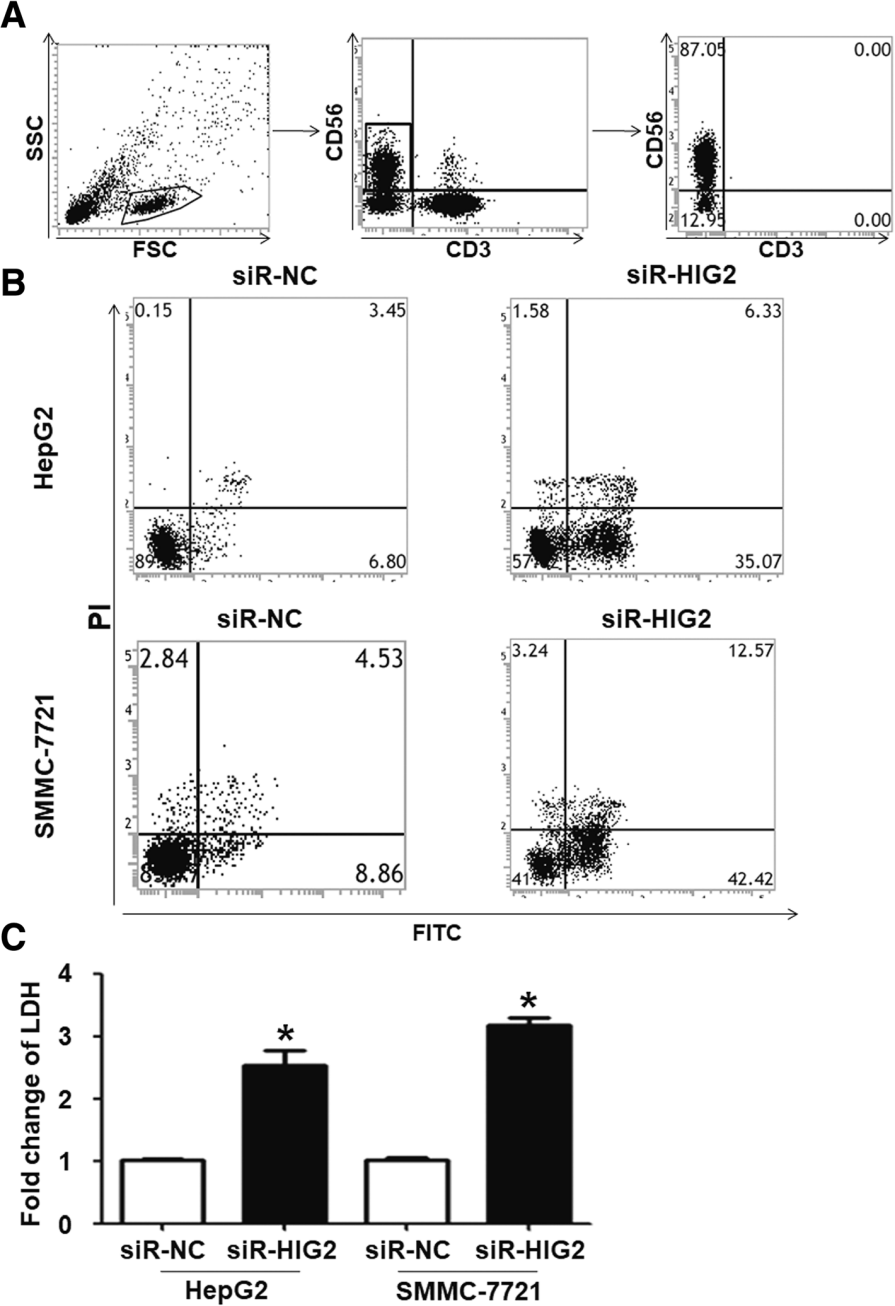
Effect of NK cells on *HIG2-*silenced HepG2 and SMMC-7721 cells. **a** Purity of NK cells higher than 90% as determined by flow cytometry. **b** Apoptosis of HepG2 and SMMC-7721 cells in the siR-HIG2 group in comparison to the siR-NC group. **c** Fold change of LDH in supernatant of HepG2 and SMMC-7721 cells in the siR-HIG2 group before and after co-culture in comparison to the siR-NC group. *P < 0.05 compared with the siR-NC group of the same cell type

To determine the effect of *HIG2* expression on the killing of HCC cells by NK cells, HepG2 and SMMC-7721 cells were transfected with siR-NC and siR-HIG2 and co-cultured with NK cells. Flow cytometry analysis showed that the purity of NK cells was more than 90%, surpassing the purity threshold required for our experiments (Fig. 4a). After being co-cultured with NK cells, apoptosis of HepG2 and SMMC-7721 cells in the siR-HIG2 group was enhanced in comparison to that in the siR-NC group (Fig. 4b). The fold change of lactate dehydrogenase (LDH) in the conditioned media of HepG2 and SMMC-7721 cells in the siR-HIG2 group before and after co-culture was significantly higher than that in the siR-NC group (*P* < 0.05) (Fig. 4c). The results suggest that the killing effect of NK cells on HepG2 and SMMC-7721 cells is enhanced after silencing *HIG2* expression in HepG2 and SMMC-7721 cells.

### Conditioned media of *HIG2-*silenced SMMC-7721 cells inhibits the phenotype and function of NK cells

**Fig. 5:**
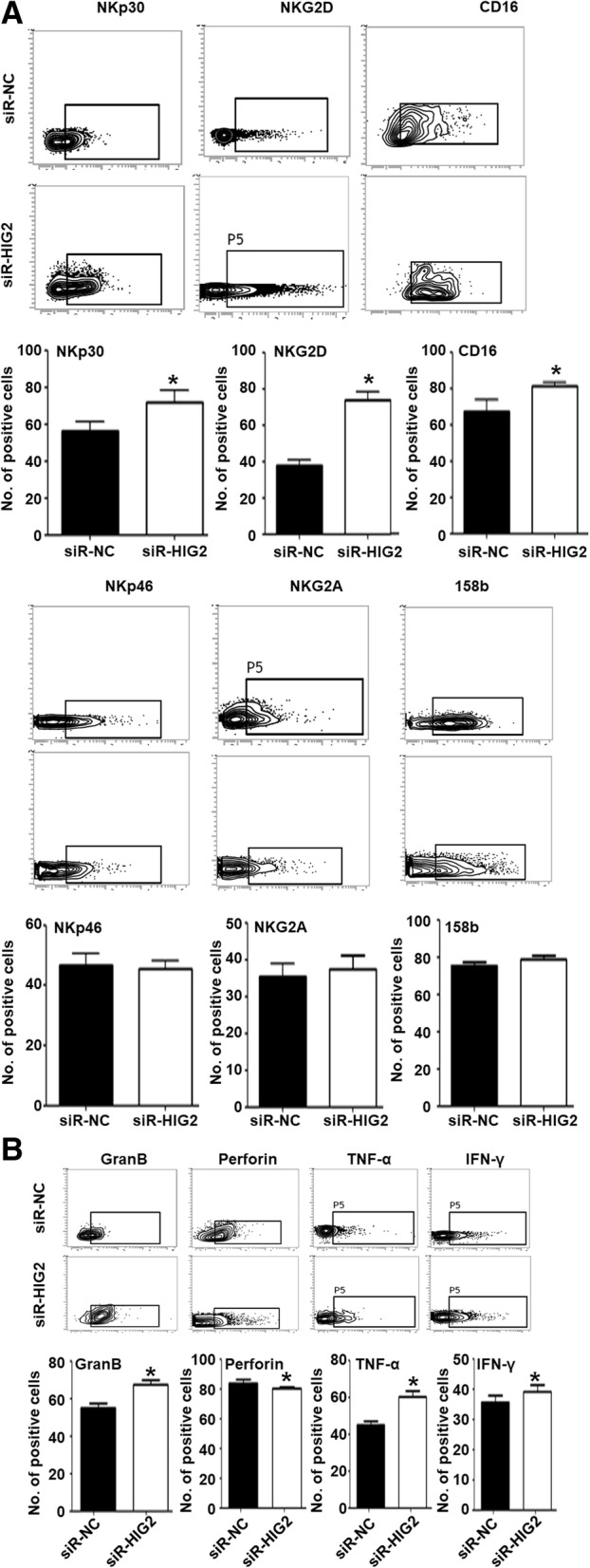
Effect of conditioned media from *HIG2-*silenced SMMC-7721 cells on the phenotype and function of NK cells. **a** Ratio of NK cells with positive expression of NKp30, NKG2D, CD16, NKp46, NKG2A, or 158b after treatment with the conditioned media of SMMC-7721 cells in the siR-HIG2 or siR-NC group. *P < 0.05 compared with the siR-NC group. **b** Ratio of NK cells with positive expression of Granzyme B, Perforin, TNF-α, or IFN-γ after treatment with the conditioned media of SMMC-7721 cells in the siR-HIG2 or siR-NC group. *P < 0.05 compared with the siR-NC group

To study the mechanism by which *HIG2* promoted the escape of HCC from killing by NK cells, we treated NK cells with the conditioned media of SMMC-7721 cells in the siR-NC and siR-HIG2 groups. Flow cytometry analysis showed that after treatment with the conditioned media of SMMC-7721 cells in the siR-HIG2 group, the proportion of NK cells with positive expression of NKG2D, NKp30, and CD16 was significantly upregulated (*P* < 0.05), while the proportion of NK cells with positive expression of NKp46, NKG2A, or 158b was not altered (*P* > 0.05) (Fig. 5a). After treatment with the conditioned media of SMMC-7721 cells in the siR-HIG2 group, the proportion of NK cells with positive expression of Granzyme B (GZMB) and TNF-α was significantly higher (*P* < 0.05), while the proportion of NK cells with positive expression of perforin or IFN-γ was not altered (*P* > 0.05) (Fig. 5b). These results indicate that of the conditioned media of *HIG2-*silenced HCC cells inhibits the phenotype and function of NK cells.

### *HIG2-*silenced HepG2 and SMMC-7721 cells modulate the activity of NK cells through the STAT3 signaling pathway

**Fig. 6:**
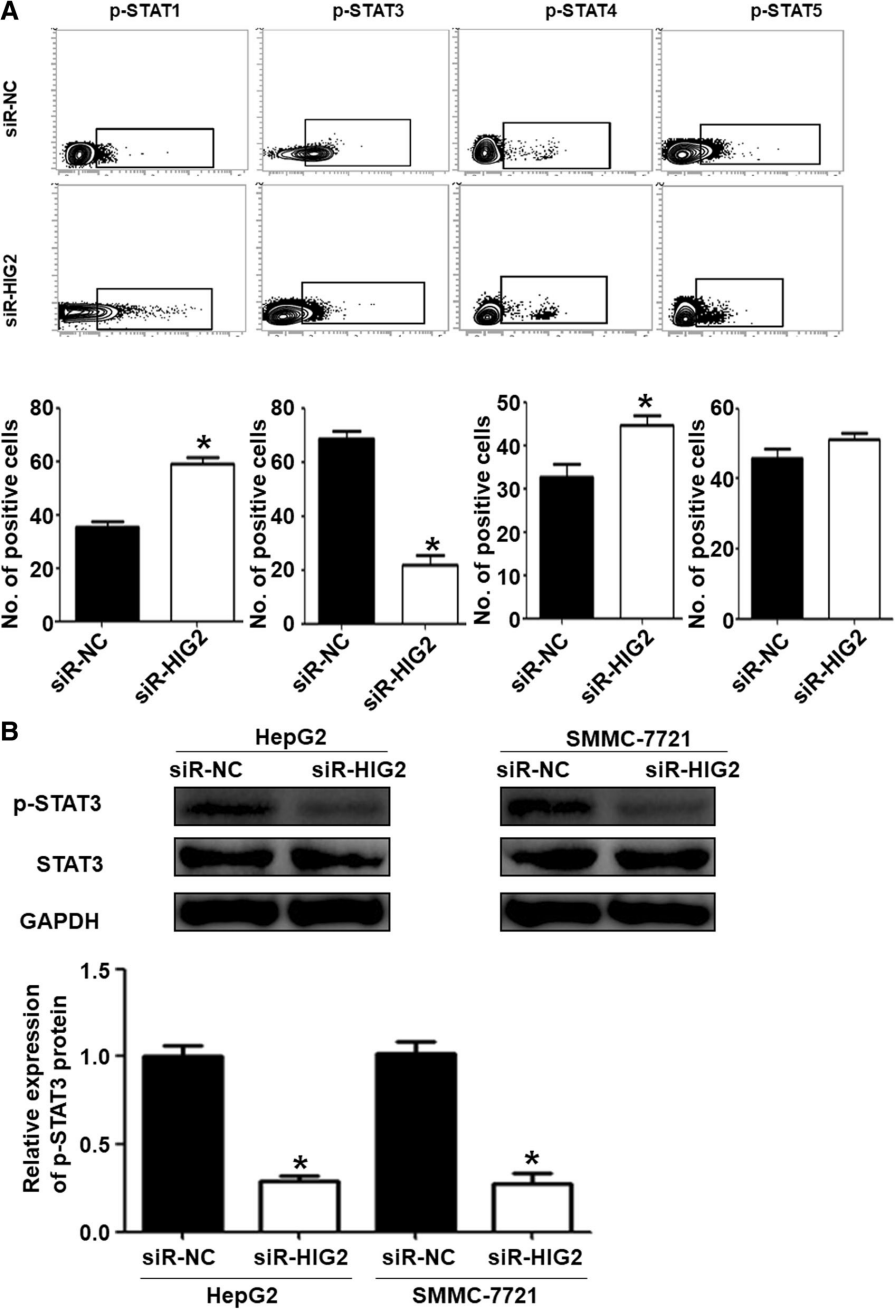
*HIG2-*silenced HepG2 and SMMC-7721 cells modulate the activity of NK cells through the STAT3 signaling pathway. **a** Phosphorylation levels of STAT1, STAT3, STAT4, and STAT5 in NK cells treated with the conditioned media of SMMC-7721 cells in the siR-HIG2 or siR-NC group, as determined by flow cytometry. *P<0.05 compared with the siR-NC group. **b** Expression of phosphorylated STAT3 protein in NK cells treated with the conditioned media of HepG2 or SMMC-7721 cells in the siR-HIG2 or siR-NC group, as determined by Western blotting. *P < 0.05 compared with siR-NC of the same cell type

To understand how *HIG2* modulates the activity of NK cells, changes in the STAT signaling pathway were examined by flow cytometry. The data showed that phosphorylation levels of STAT1 and STAT4 in NK cells treated with the conditioned media of SMMC-7721 cells in the siR-HIG2 group were significantly higher than those in the siR-NC group (*P* < 0.05). The phosphorylation level of STAT3 in NK cells treated with the conditioned media of SMMC-7721 cells in the siR-HIG2 group was significantly lower than that in the siR-NC group (*P* < 0.05). Additionally, the phosphorylation level of STAT5 in NK cells treated with the conditioned media of SMMC-7721 cells in the siR-HIG2 group was not significantly different from that in the siR-NC group (*P* > 0.05) (Fig. 6a). Western blotting showed that the expression of phosphorylated STAT3 in NK cells treated with the conditioned media of HepG2 or SMMC-7721 cells in the siR-HIG2 group was significantly lower than that in the siR-NC group (*P* < 0.05) (Fig. 6b). The results suggest that *HIG2-*silenced HepG2 and SMMC-7721 cells can modulate the activity of NK cells through the STAT3 signaling pathway.

### *HIG2* gene promotes the evasion of HCC cells from killing by NK cells through upregulation of IL-10

**Fig. 7:**
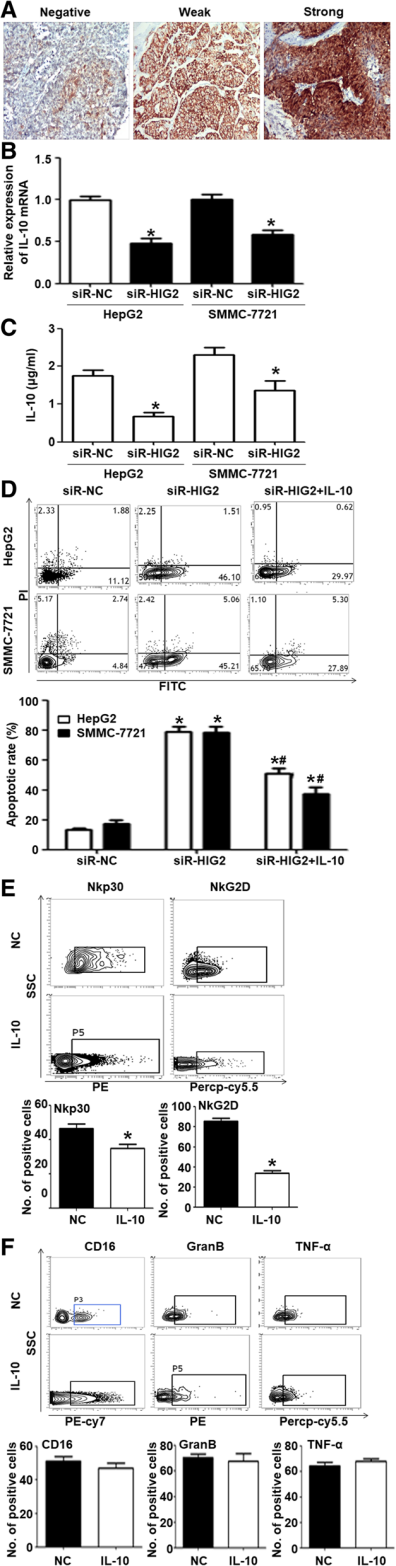
*HIG2* gene promotes the evasion of HCC cells from killing by NK cells through upregulation of IL-10 expression. **a** IL-10 protein expression in HCC tissues or tumor-adjacent tissues, as determined by immunohistochemistry. **b** and **c** *IL-10* mRNA expression or secreted IL-10 protein in HepG2 and SMMC-7721 cells transfected with siR-HIG2 or siR-NC, as determined by qRT-PCR and ELISA, respectively. *P < 0.05 compared with the siR-NC group of the same cell type. **d** Apoptotic rates of HepG2 and SMMC-7721 cells in the siR-NC, siR-HIG2, and siR-HIG2 + IL-10 groups. *P < 0.05 compared with the siR-NC group; #*P* < 0.05 compared with the siR-HIG2 + IL-10 group. **e** Proportion of NK cells with positive expression of NKp30 and NKG2D receptors after treatment with IL-10. *P < 0.05 compared with the NC group. **f** Proportion of NK cells with positive expression of CD16, GZMB, or TNF-α after treatment with IL-10

Increasing evidence revealed that IL10 is one of the key negative regulators of NK cell activity through STAT3 pathway. To test whether *HIG2* can help HCC cells escape immune surveillance of NK cells through IL-10, we co-cultured NK cells with IL-10-containing conditioned media of HepG2 and SMMC-7721 cells. Immunohistochemistry analysis showed that IL-10 protein expression in HCC tissues was higher than that in tumor-adjacent tissues (Fig. 7a). qRT-PCR and ELISA data showed that *IL-10* mRNA expression in HepG2 and SMMC-7721 cells transfected with siR-HIG2 was significantly lower than that in the siR-NC group (*P* < 0.05) (Fig. 7b and c). Flow cytometry analysis showed that the apoptotic rates of HepG2 and SMMC-7721 cells in the siR-HIG2 + IL-10 group were significantly higher than those in the siR-NC group (*P* < 0.05) but were significantly lower than those in the siR-HIG2 group (*P* < 0.05) (Fig. 7d). After treatment with IL-10, the proportion of NK cells with positive expression of NKp30 and NKG2D receptors was significantly higher than that of the NC group (*P* < 0.05) (Fig. 7e), but the proportion of NK cells with positive expression of CD16, GZMB, or TNF-α was not significantly different from that of the NC group (*P* > 0.05) (Fig. 7f). The results indicate that *HIG2* promotes the evasion of HCC cells from killing by NK cells through upregulation of IL-10.

### *HIG2* promotes IL-10 expression through the AMPK/CREB signaling pathway

**Fig. 8:**
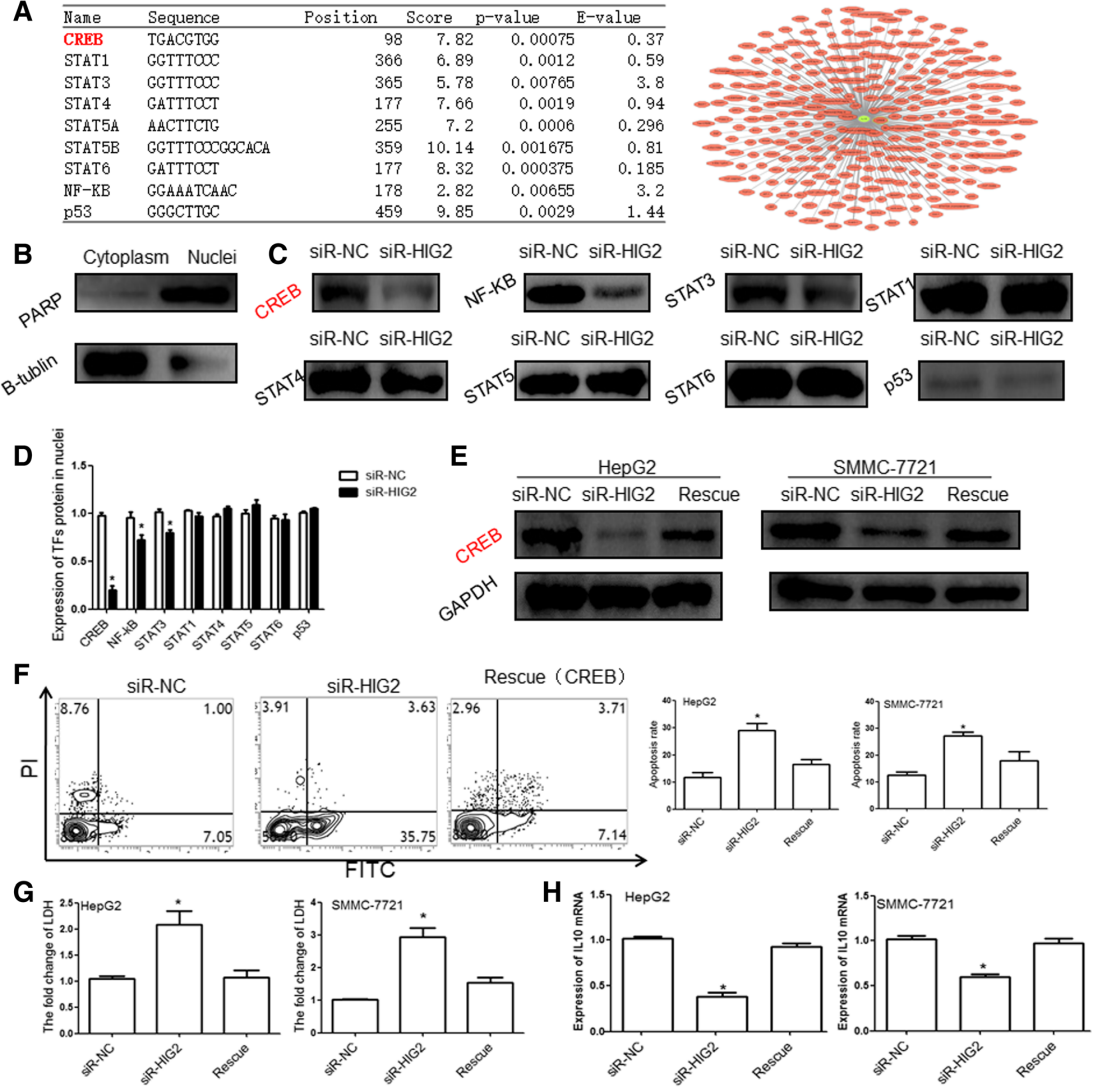
**a** Indicated transcription factors (TFs) may directly bind to the promoter of the *IL-10* gene. **b-d** Expression of the indicated TFs in nuclei of indicated HCC cells by Western blotting. **e** Expression of CREB protein in *HIG2-*silenced HCC cells (**e**). **f–h** Flow cytometry, LDH detection and Qrt-pcr were performed to analyse the restoration of CREB for the phenotype of *HIG2-*silenced HCC cells or the expression of IL-10

**Fig. 9:**
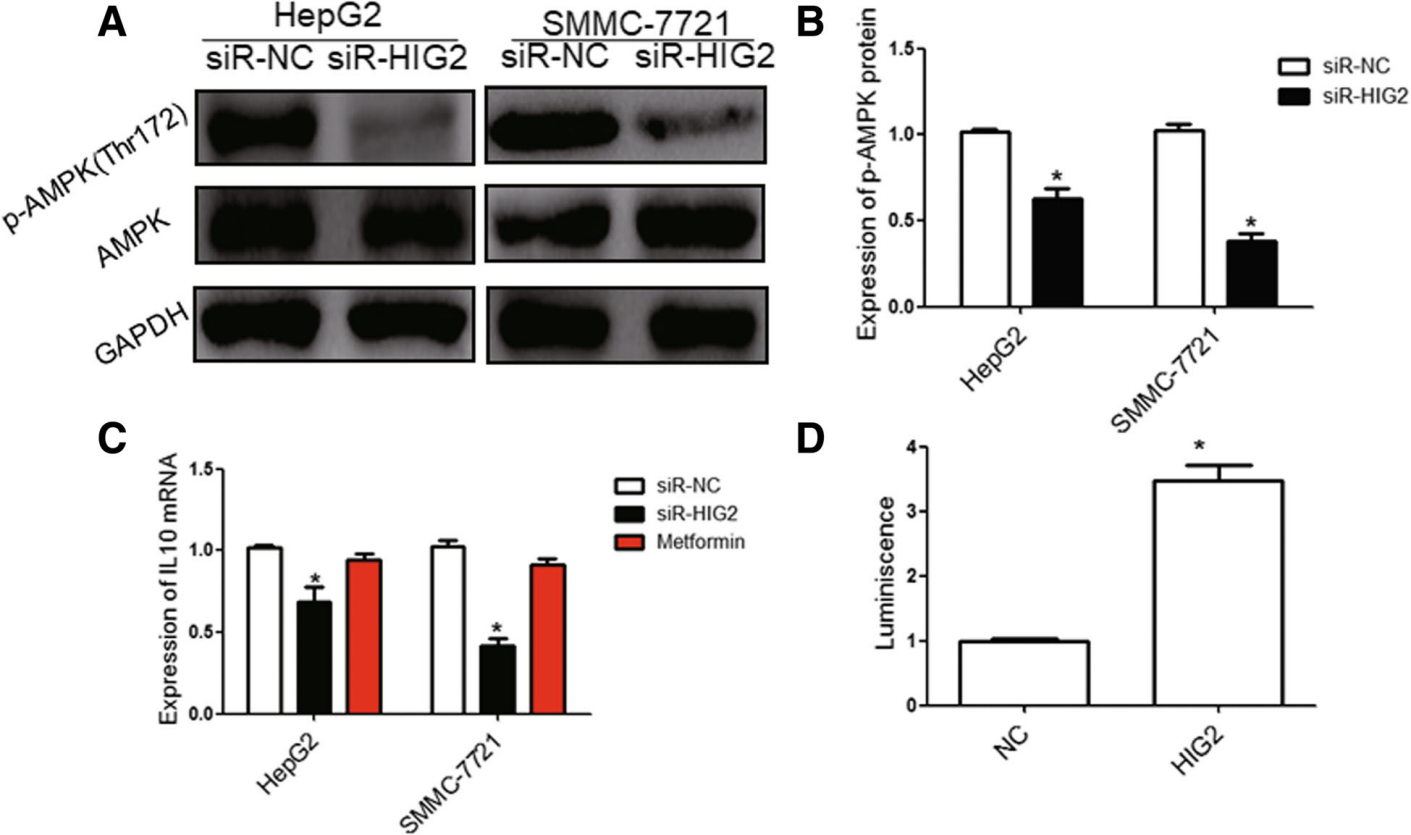
**a-b** Expression of phospho-AMPKα (Thr172) in *HIG2-*silenced HCC cells. **c** Effect of metformin hydrochloride, an activator of AMPK signaling, on IL-10 expression in *HIG2-*silenced HCC cells. **d** Dual luciferase reporter assay was performed to detect the change of transcriptional activity of IL-10, *P < 0.05 compared with the NC group

To further investigate the underlying mechanism by which *HIG2* regulates IL-10 expression in HCC cells, we performed bioinformatic analysis to identify the signaling pathway that regulates IL-10 expression. Our results revealed that several transcription factors (TFs), which are involved in the AMPK, NF-kB, and STAT signaling pathways, may directly bind to the promoter of the *IL-10* gene (Fig. 8a). Furthermore, we determined the expression of the TFs in the nuclei of the indicated HCC cells by Western blotting and found that CREB expression was significantly inhibited in the nuclei of *HIG2-*silenced HCC cells. However, the expression of other TFs showed no significant changes (Fig. 8b-d). Next, we overexpressed CREB protein in *HIG2-*silenced HCC cells (Fig. 8e) and found that CREB not only restored the phenotype of *HIG2-*silenced HCC cells but also increased the expression of IL-10 (Fig. 8f-h). These observations indicate that HIG2 regulated IL-10 expression via CREB. CREB is a well-known downstream factor of AMPK signaling. Therefore, we investigated the activation of the AMPK pathway. The results revealed that phospho-AMPKα (Thr172) was suppressed in *HIG2-*silenced HCC cells (Fig. 9a and b). Additionally, we confirmed that metformin hydrochloride, an activator of AMPK signaling, could restore IL-10 expression in *HIG2-*silenced HCC cells (Fig. 9c). Dual luciferase reporter assay also revealed that CREB protein could enhance the transcriptional activity of IL-10 (Fig. 9d). These results indicate that the *HIG2* gene promotes IL-10 expression through the AMPK/CREB signaling pathway.

## Discussion

At present, recurrence and metastasis are key factors that limit clinical outcomes of HCC patients [3]. Immune escape is also an important prerequisite for tumor recurrence and metastasis [19]. NK cells are important cells in the innate immune system, and they can quickly identify and kill tumor cells [18, 20]. The activation of multiple oncogenes increases the metastatic ability of tumor cells, but the effect of these genes on the immunological properties of tumor cells is unclear.

As a specific downstream target gene of HIF-1, *HIG2* plays an important role in the activation of the hypoxia-induced signaling pathway, which is closely related to the proliferation and metastasis of tumor cells [21]. Using bioinformatics, we discovered that the overall and disease-free survival rates of HCC patients with high expression of *HIG2* were significantly lower than those of HCC patients with low expression of *HIG2*, indicating that the expression level of *HIG2* is of clinical significance for the prognosis of HCC patients. qRT-PCR results showed that the expression of *HIG2* was significantly upregulated in HCC tissues, and positively correlated with lymph node metastasis and TNM stage, suggesting that *HIG2* is associated with the occurrence and development of HCC. Immunohistochemical analysis showed that the positive expression of HIG2 protein in HCC tissues was significantly higher than that in tumor-adjacent tissues. Elevated levels of HIG2 were also observed in HCC cell lines HepG2 and SMMC-7221. After interfering with the expression of *HIG2* in HepG2 and SMMC-7721 cells, the proliferation, migration, and invasion were significantly inhibited, indicating that *HIG2* may function as an oncogene in HCC.

Increasing evidence shows that numerous lymphocytes infiltrate tumor tissues to inhibit tumor growth and metastasis [22, 23]. Therefore, evading killing by immune cells is one of the key factors driving tumor cell survival. Tumor cells can promote immune evasion by altering their immunogenicity and regulating the activity of immune cells [24]. For example, tumor cells can escape killing by NK cells through autocrine downregulation of the expression of MICA/B protein [25]. In addition, tumor cells can induce macrophage differentiation into tumor associated macrophage type 2 (TAM2), thereby promoting tumor cell immune escape [26]. In the present study, after interfering with *HIG2* gene expression, the killing effect of NK cells on HCC cells was significantly enhanced. There was also an appreciable fold increase in LDH release from *HIG2*-silenced cells in comparison with controls, suggesting that *HIG2* helps HCC cells escape NK cell-mediated cytotoxicity. Our flow cytometry results showed that conditioned media of *HIG2-*silenced HCC cells stimulated the expression of the activated receptor NKp30, NKG2D, and CD16 on NK cells, and upregulated the expression of the effector molecules GZMB, Perforin, TNF-α, and IFN-γ. These data suggest that *HIG2-*silenced HCC cells can enhance the killing activity of NK cells.

The STAT signaling pathway plays an important regulatory role in NK cell differentiation, maturation, and activation. For example, STAT1 and STAT2 can activate NK cells, while STAT3 inhibits NK cell activity [27, 28]. Our results showed that the conditioned media of *HIG2-*silenced HCC cells significantly reduced the phosphorylation level of STAT3, but only slightly elevated the phosphorylation levels of STAT1 and STAT4 proteins, which can promote the activity of NK cells. Therefore, we hypothesize that the conditioned media of *HIG2-*silenced HCC cells can upregulate the activity of NK cells by inhibiting intracellular STAT3 signaling.

Studies have shown that the STAT3 signaling pathway in NK cells is regulated by many cytokines such as IL10, IL-12, and IL-15 [29]. Among these, the effect of IL-10 on the activation of the STAT3 pathway is the most significant. In the present study, we found that IL-10 protein expression in HCC tissues was significantly higher than that in tumor-adjacent tissues, and *IL-10* mRNA levels in *HIG2-*silenced HCC cells were significantly decreased. Additionally, treatment with IL-10 protein significantly restored the cytotoxic capacity of NK cells, which had been inhibited by *HIG2-*silenced HCC cells. Flow cytometry showed that treatment with an IL-10 antibody significantly up-regulated the expression of activated receptors NKp30 and NKG2D on the surface of NK cells and down-regulated the expression of p-STAT3 in NK cells, suggesting that *HIG2* induced the activation of the STAT3 signaling pathway in NK cells by up-regulation of IL-10 expression in HCC cells. Consequently, the tumor killing activity of NK cells was reduced, promoting the metastasis of HCC cells. Mechanistically, we found that CREB can enhance the transcriptional activity of IL-10 and confirmed that HIG2 can increase the expression of p-AMPKα and CREB nuclear import. These data indicate that HIG2 can increase IL-10 expression through the AMPK/CREB signaling pathway.

## Conclusion

The present study demonstrates that the *HIG2* gene is highly expressed in HCC and is closely related to tumor progression and prognosis. Mechanistically, HIG2 increases IL-10 expression via AMPK/CREB signaling, and the secreted IL-10 inhibits the cytotoxicity of NK cells through the STAT3 signaling pathway, thereby promoting the recurrence and metastasis of HCC.

## Abbreviations

DAB: Diaminobenzidine
FBS: Fetal bovine serum
GEPIA: Gene Expression Profiling Interactive Analysis
HCC: Hepatocellular carcinoma
HIF-1: Hypoxia-inducible factor-1
HIG2: Hypoxia-inducible gene 2
LD: Lipid droplet
LDH: Lactate dehydrogenase
NK: Natural killer
PBS: Phosphate-buffered saline
qRT-PCR: Quantitative real-time polymerase chain reaction
